# On the Absorption and Elimination of Quinine

**Published:** 1881-07

**Authors:** 


On the Absorption and Elimination of Quinine.—From a series of experiments undertaken on this point, Professor Lepidichioti, (*Il Morgagni; La Presse Med. Belge*) concludes: 1. Quinine is certainly not eliminated by the saliva; 2. It is not eliminated by the sweat; 3. It is not absorbed after friction upon the skin; 4. It appears at the end of thirteen to fifteen minutes in urine when it has been administered by hypodermic injection; 5. It appears after fifteen to seventeen minutes when it has been administered by the mouth and the primæ viæ are in good condition; 6. It is preceptible at the end of twenty to twenty-five minutes when it has been administered by means of the entero-clysm, and the patient has retained this for some time; 7. It is found at the end of thirty to forty minutes when it has been injected by the aid of the ordinary clysopompe (Davidson's syringe), and has been retained some time.—*St. Louis Courier of Medicine.*

